# *In silico* assessment of diterpenes as potential inhibitors of SARS-COV-2 main protease

**DOI:** 10.2217/fvl-2022-0163

**Published:** 2023-05-22

**Authors:** Yousef A Abdelrady, Naeem Mahmood Ashraf, Arslan Hamid, Hayam S Thabet, Asmaa M Sayed, Shimaa H Salem, Emad HM Hassanein, Ahmed M Sayed

**Affiliations:** 1 Faculty of Pharmacy, Assiut University, Assiut, 71526, Egypt; 2 School of Biochemistry & Biotechnology, University of the Punjab, Lahore, Pakistan; 3 LIMES Institute (AG-Netea), University of Bonn, Bonn, Germany; 4 Microbiology department, Faculty of Veterinary Medicine, Assiut University, 71526, Egypt; 5 Botany & Microbiology Department, Faculty of Science, Assiut University, Egypt; 6 Department of Pharmacology & Toxicology, Faculty of Pharmacy, Al-Azhar University, Assiut Branch, Assiut, 71524, Egypt; 7 Biochemistry Laboratory, Chemistry Department, Faculty of Science, Assiut University, 71516, Egypt

**Keywords:** diterpenes, inhibitors, molecular docking, Mpro, SARS-CoV-2

## Abstract

**Aim:**

We aimed to investigate the potential inhibitory effects of diterpenes on SARS-CoV-2 main protease (Mpro).

**Materials & methods:**

We performed a virtual screening of diterpenoids against Mpro using molecular docking, molecular dynamics simulation and absorption, distribution, metabolism and excretion) analysis.

**Results:**

Some tested compounds followed Lipinski’s rule and showed drug-like properties. Some diterpenoids possessed remarkable binding affinities with SARS-CoV-2 Mpro and drug-like pharmacokinetic properties. Three derivatives exhibited structural deviations lower than 1 Å.

**Conclusion:**

The findings of the study suggest that some of the diterpenes could be candidates as potential inhibitors for Mpro of SARS-CoV-2.

Coronaviruses are a broad family of enveloped viruses characterized by positive-sense, large, ssRNA genomes, including Middle East respiratory syndrome coronavirus, SARS-CoV-1 and 2 [1]. SARS-CoV-2 is an extremely contagious virus that has caused over 4 million deaths, the second most consequential health crisis after the influenza pandemic of 1918 [2]. Viral structural proteins, such as spike, envelope, membrane and nucleocapsid, translation and activation are independent of the viral proteases. On the contrary, nonstructural viral proteins (nsps) are not active until they are cleaved by viral proteases into functional replication and transcription factors such as nsp12 or RNA-dependent-RNA polymerase and nsp13 or helicase [3]. Main protease (Mpro), spike protein, nsp12, nsp13 and papain-like protease (PLpro) are considered promising targets for antiviral agents. However, the PLpro has a similar recognition site as deubiquitinases, normally found in humans, which may cause unfavorable effects [4–6]. SARS-CoV-2 spike protein shows only 75% sequence homology with SARS-COV spike protein [7,8]. SARS-COV-1 and 2 nsp12 are 96% homologous [9]. SARS-CoV-1 and 2 nsp13 have 98.5% sequence similarity [10]. Although nsp12 and nsp13 have high sequence homology in both virus strains, their activation requires pre-hydrolysis of the viral polyproteins by the Mpro setting SARS-CoV-2 Mpro as the primary target for potential antiviral agents. Diterpenes comprise a wide class of natural products with remarkable biological activities, including antibacterial, antifungal, antitumor and cardiovascular effects [11]. They inhibited the replication of several viruses, such as SARS-COV, Chikungunya virus (CHIKV), HIV-1,2 and HSV-2 through various mechanisms. Supplementary Table 1 summarizes the antiviral activity of some diterpenoids and their mechanisms of action. It was reported that abietane derivatives have an inhibitory effect on SARS-CoV Mpro [12,13]. Jatrophane derivatives inhibit the replication of CHIKV, HIV-1,2, Semliki Forest virus (SFV) and Sindbis viruses by modulating the protein kinase C isozymes [14]. Additionally, segetane and paraliane derivatives suppress HIV-1 replication [15] and pepluane derivatives inhibit HSV-2 [16]. According to previous studies, SARS-CoV-1 and 2 Mpro sequences showed a high sequence homology of 96–97%. Therefore, we studied the binding affinities of diterpenes with SARS-CoV-2 Mpro. The chemical structures of diterpenoid skeletons are shown in Supplementary Figure 1. A molecular docking approach was used to anticipate the binding affinities of diterpenoids with SARS-CoV-2 Mpro. Some classes showed higher binding affinities than SARS-CoV-2 Mpro inhibitor, GC373, which is a potent SARS-CoV-2 Mpro inhibitor and a promising drug candidate for COVID-19 [17,18]. This systematic study provides insights into the binding energies and interaction modes of diterpenes with SARS-CoV-2 Mpro to be evaluated for the future clinical trials.

## Materials & methods

### Sequence alignment

The amino acid sequence of the Mpro of SARS-CoV and SARS-CoV-2 were obtained from the National Center for Biotechnology Information (NCBI GenBank) with protein accession nos. ATO98144.1 and YP_009742612.1, respectively. Sequence alignment was performed using the MUSCLE server (https://www.ebi.ac.uk/Tools/msa/muscle/).

### Molecular docking

The Autodock vina1.5.6 was used to perform the molecular docking of diterpenes against SARS-CoV-2 Mpro. The crystal structure of Mpro with its inhibitor GC373 (Protein Data Bank [PDB] ID: 6WTJ) was retrieved from the PDB. The inhibitor structure and the water molecules were removed from the PDB 3D structure, leaving only polar hydrogens. The experimental binding inhibitors and the binding pocket of Mpro were identified according to the available crystal structures [19]. The average of the lowest energy of docking was used to outline the binding affinity with Mpro. The best-scored confirmation has been chosen and visually analysed using Chimera 1.12 software. The relevant experimental binding ligand was redocked into Mpro, and its energy score of docking was used to evaluate the predicted binding affinity of the various diterpenes with Mpro.

### Docking validation

PDB structure of GC373 was prepared by MarvinJS and docked into the active site of Mpro using the same steps and grid parameters. The root mean square deviation (RMSD) was estimated by the superimposition of the redocked complex on the co-crystallized complex using Pymol 2.3 and the amino acid interactions in both complexes were visualized and compared using ligplot +v.2.2.

### ADME analysis & drug likeness

To test whether the compounds are good drug candidates, a pharmacokinetics study was performed using the Swiss absorption, distribution, metabolism and excretion (ADME) web tool (http://www.swissadme.ch/). The chemical structures were drawn using MarvinJs.

### Molecular dynamics simulation analysis

Molecular dynamics simulations (MDS) of five docked complexes were performed using GROMACS 5.0.5 [20]. The protein topology file was prepared using CHARMM-36 forcefield [21]. Ligands topology files were prepared using the CGenFF server [22]. The system was solvated using a TIP3P water system and neutralized using appropriate ions. The energy minimization was carried out using a decent gradient algorithm [23]. The system was equilibrated using constant temperature, constant volume (NVT) and constant temperature, constant pressure (NPT) ensembles for 100 ps. Finally, the 30 ns molecular dynamics (MD) production phase was performed. The MD trajectory was analyzed to calculate the RMSD of the ligand regarding the binding site. Similarly, root means square fluctuation of protein C-alpha was calculated to observe structural fluctuations in the protein upon binding of ligand. Furthermore, the total number of hydrogen (Hb) bonds between ligands and the protein were calculated for a 30 ns period. Finally, Coul-SR and LJ-SR interaction energies were calculated to measure the strength of ligands and the protein binding [24].

MMGBSA method, implemented in Amber 10 was used to calculate the binding energies (kcal/mol) of ligands (peptide constructs) with protein using MD trajectories [25,26]. For each complex, the binding free energies were calculated using the following equation:

(Equation 1) $$\Delta G_{\text{bind}}=G_{\text{complex}}-G_{\text{protein}}-G_{\text{ligand}}$$

In Equation 1, Δ*G*_bind_ represents the binding free energy, while *G*_complex_ is the overall free energy of ligand and protein complex. *G*_protein_ and *G*_ligand_ are the individual free energies of protein and ligands, respectively. The free energies are calculated using the following equation:

(Equation 2) $$\Delta E_{\text{MM}}+\Delta G_{\text{GB}}+\Delta G_{\text{nonpolar}}-T\Delta S$$

In Equation 2, Δ*E*_MM_ is the binding energy between Mpro and ligand in gas-phase. Δ*G*_GB_ and Δ*G*_nonpolar_ are desolvation free energy of polar and non polar components of system, respectively, while *T*Δ*S* is the change of entropy on the binding of the ligand with the protein.

## Results

### Sequence homology between SARS-CoV-1 & 2 Mpro

The similarity percentages and sequence alignment were conducted between SARS-COV (PDB ID: 7EO8) and SARS-CoV-2 (PDB ID: 6wtj) Mpros and 96% sequence homology was observed (Supplementary Figure 2); in addition to the catalytic dyad, other key amino acids including His41, Met49, phe140, Leu141, Asn142, Gly143, Ser144, Cys145, His163, His164, Met165, Glu166 and His172, are conserved in both proteins.

### Diterpenes as potential SARS-CoV-2 Mpro inhibitors

Binding affinities and interaction modes of various diterpenoids with SARS-CoV-2 Mpro were evaluated using the Autodock Vina software, a molecular docking tool used for computational studies. Our results showed that abietane, jatrophane, segetane. pepluane, paraliane derivatives (Supplementary Figure 3) have higher binding affinities with SARS-CoV-2 Mpro than other classes of diterpenes (Table 1). Derivatives of these classes showing reported antiviral activities were docked with SARS-CoV-2 Mpro. Docking results of diterpenoids were compared with the results obtained from docking a SARS-CoV-2 Mpro inhibitor, GC373, which has a high binding affinity with SARS-CoV-2 Mpro and cocrystallize with the protein as a stable complex (PDB: 6WTJ). The binding energies of the selected derivatives with SARS-CoV-2 are listed in Table 2. The results show that six derivatives (5, 13, 14, 21, 22, 24) have higher binding affinities (-6.63, -6.62, -6.59, -6.73, -6.58, -6.71 kcal/mol, respectively) than the redocked standard (-6.52 kcal/mol), which highly suggest the formation of stable complexes with Mpro and emphasizes their potential inhibitory role.

**Table 1.** Binding affinities of some classes of diterpenes skeletons with SARS-CoV-2 main protease.

Compound	Lowest energy of docking (kcal/mol)
Labdane	-5.34 ± 0.34
Clerodane	-5.84 ± 0.24
Abietane	-6.03 ± 0.57
Totarane	-5.97 ± 0.34
Pimarane	-5.93 ± 0.37
Fujinane	-5.68 ± 0.25
Tigliane	-5.97 ± 0.25
Taxane	-5.84 ± 0.35
Jatrophane	-6.21 ± 0.28
Segetane	-6.77 ± 0.46
Pepluane	-6.62 ± 0.44
Paraliane	-6.74 ± 0.28
GC373 (Standard)	-6.52 ± 0.30

**Table 2.** Binding energies of abietane, jatrophane, segetane, pepluane, paraliane derivatives and GC373.

No.	Lowest energy of docking (kcal/mol)
1^†^	-6.24 ± 0.36
2^†^	-6.4 ± 0.47
3^†^	-6.20 ± 0.29
4^†^	-6.23 ± 0.50
5^†^	-6.63 ± 0.38
6^‡^	-5.42 ± 0.33
7^‡^	-5.48 ± 0.24
8^‡^	-6.00 ± 0.28
9^‡^	-5.90 ± 0.45
10^‡^	-5.27 ± 0.41
11^‡^	-5.52 ± 0.23
12^‡^	-5.53 ± 0.26
13^‡^	-6.72 ± 0.22
14^‡^	-6.59 ± 0.39
15^‡^	-5.78 ± 0.32
16^‡^	-6.00 ± 0.47
17^§^	-5.39 ± 0.21
18^§^	-6.36 ± 0.41
19^§^	-6.33 ± 0.34
20^§^	-6.02 ± 0.41
21^§^	-6.73 ± 0.47
22^¶^	-6.58 ± 0.50
23^#^	-6.26 ± 0.65
24^#^	-6.71 ± 0.63
Standard	-6.52 ± 0.30

† Abietane

‡ Jatrophane

§ Segetane

¶ Pepluane

# Paraliane

**Figure 1.**
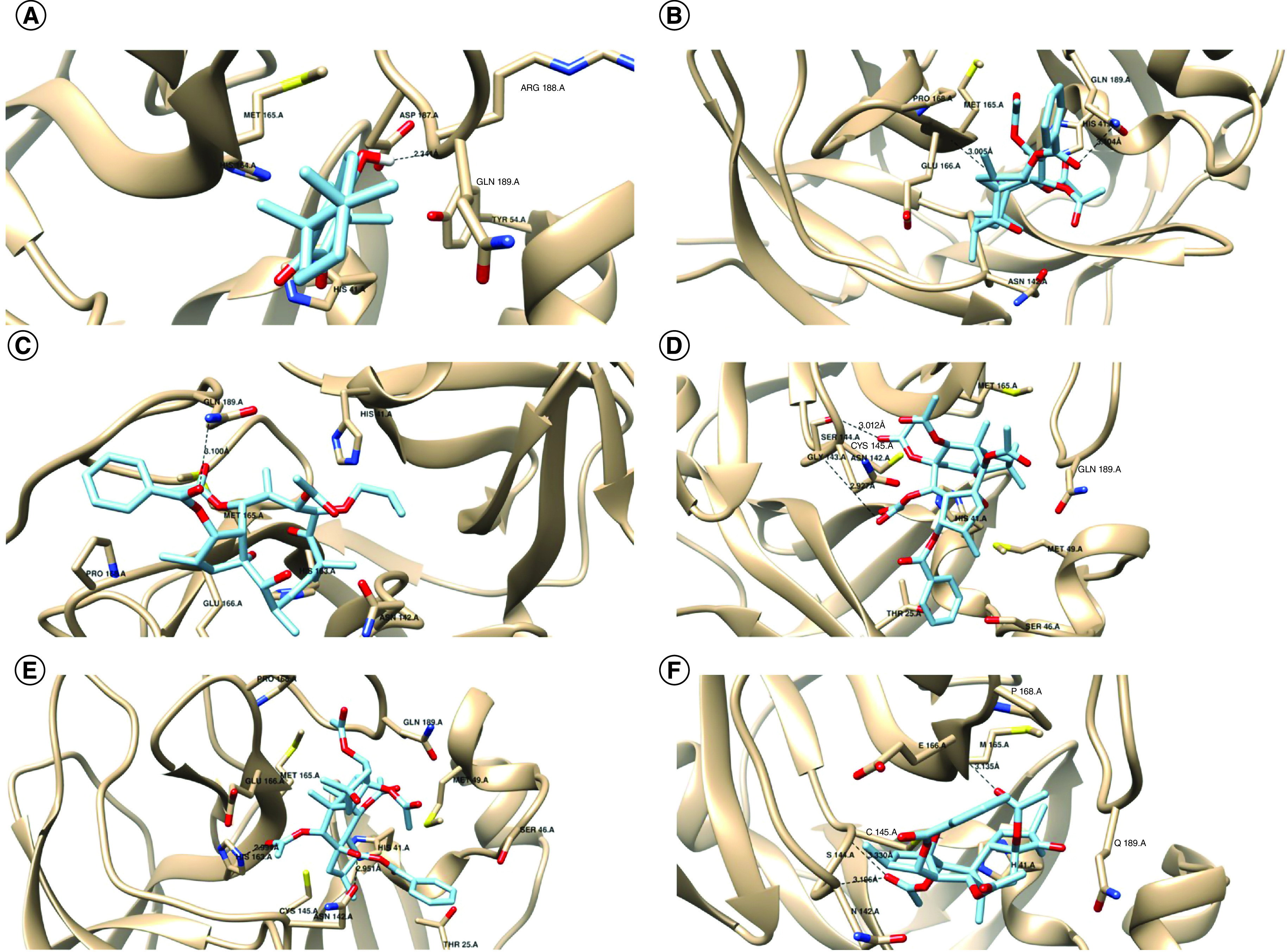
A 3D representation of interactions between the most promising derivatives in each class and the key amino acids of SARS-CoV-2 main protease. **(A)** Compound 5. **(B)** Compound 13. **(C)** Compound 14. **(D)** Compound 21. **(E)** Compound 22. **(F)** Compound 24.

**Figure 2.**
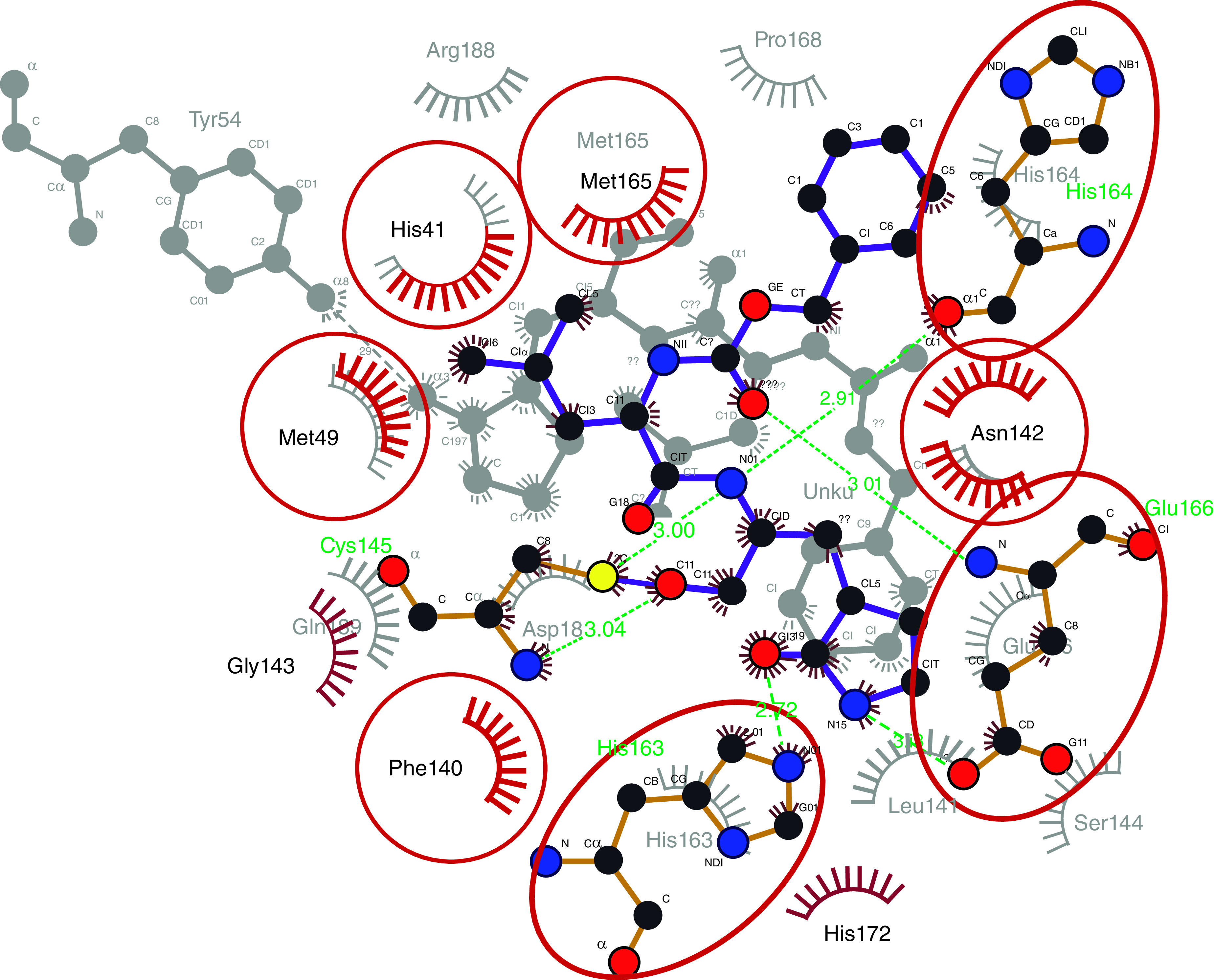
Superimposed amino acids of the redocked complex GC373-main protease (gray) on the co-crystallized complex (violet-red) using Ligplot.

The interaction modes of the high-scoring derivatives with Mpro are illustrated as 3D images in Figure 1. Unique structural features in diterpenoids, including the polar groups (OH, OAc, C=O) and the aromatic rings (OBz) enable them to form multiple non-covalent interactions with the binding site of the target protein. Derivative 5 makes hydrophobic interaction with H41, M165 and the hydroxyl group on C1 forms a 2.3 Å H-bond with R188 (Figure 1A). Derivative 13 carbonyl group on C14 forms 3.1 Å H-Bond with E166 and carbonyl of the benzoic group on C3 forms 3.0 Å H-bond with Q189. Also, there is aromatic–proline interaction between the aromatic ring of compound 13 and P168 of the active site CH/π interaction (Figure 1B). Derivative 14 carbonyl of the benzoic group on C1 forms a 3.1 Å-hydrogen bond with the side chain of Q189 and CH/π interaction with P168 (Figure 1C). Derivative 21 carbonyl of the acetoxy group on C4 forms a 2.9 Å H-bond with G143, and the carbonyl of the acetoxy group on C9 forms a 3.0 Å H-bond with S144 (Figure 1D). Derivative 22 hydroxyl group on C1 forms 2.9 Å H-bond with the side chain of N142 and carbonyl of the acetoxy group on C9 forms 2.9 Å H-bond with H163 (Figure 1E). Derivative 24 undergoes CH/π interaction with P168. The carbonyl of the acetoxy group on C9 forms 3.1 Å H-bond with S144 and 3.3 Å H-bond with C145. The carbonyl of the acetoxy group on C11 forms 3.1 Å H-bond with M165 (Figure 1F). Based on the interaction modes and strengths, derivatives 21 and 24 show the most stabilized complexes with the protein as they form H-bonds with the oxyanion hole residues G143, S144 and C145, respectively, while derivative 5 forms the shortest H-bond with R188. Docking results suggest that diterpenoids have similar or higher potential binding affinities with SARS-CoV-2 Mpro than the standard. Since GC373 inhibits the protein with IC50 of 0.40 ± 0.05 μM [18], diterpenes are expected to possess the potential inhibitory effect on SARS-CoV-2 Mpro. To verify our docking results, RMSD calculation and superimposition of the redocked ligand on the co-crystallized ligand were applied. RMSD was calculated by superimposing the redocked complex on the co-crystallized complex, which was 0.829 Å, within the success range (Supplementary Figure 4) [27]. The interacting amino acids in both the redocked complex and the experimental complex were observed in Ligplot +v.2.2. and found to be the same (His41, Met49, Phe140, Asn142, His163, Glu166). In addition, the interaction modes are conserved in both structures. The superimposed amino acids are highlighted with red circles in the 2D illustration (Figure 2).

### ADME analysis

The pharmacokinetic properties of the derivatives that achieved the best docking results were anticipated using SwissADME online free tool, and the results are listed in Table 3. Five of the tested compounds (1, 2, 3, 4, 5) are supposed to be possible drugs as they follow Lipinski’s rule and have TPSA less than 131.6 Å2 and WLOGP less than 5.88.

**Table 3.** Predicted pharmacokinetic properties of the best-scored diterpenes.

Compounds	Molecular weight	H-bond acceptor	H-bond donor	MLogP	Lipinski’s violation	TPSA (Å^2^)	WLOGP
1^†^	286.45	1	1	4.92	1	20.23	5.55
2^†^	284.44	1	0	4.56	1	17.07	5.48
3^†^	300.44	2	1	3.9	0	37.30	5.19
4^†^	302.45	2	1	3.73	0	37.3	4.44
5^†^	314.42	3	1	2.67	0	54.37	4.31
8^‡^	648.69	13	1	1.08	2	185.87	2.66
9^‡^	608.63	13	1	0.59	2	185.87	1.71
13^‡^	552.61	11	2	1.04	2	162.73	1.82
14^‡^	624.80	8	0	4.33	2	105.20	6.77
16^‡^	614.68	11	2	2.04	2	162.73	3.11
17^§^	592.63	12	1	1.49	2	168.80	2.12
18^§^	696.78	12	1	2.99	2	168.80	4.15
19^§^	714.75	14	1	2.13	2	195.10	2.89
20^§^	730.75	15	2	1.40	2	215.33	1.87
21^§^	642.73	11	2	2.55	2	162.73	3.42
22^¶^	658.73	12	2	2.61	2	171.96	3.29
23^#^	656.72	12	1	2.53	2	168.80	3.35
24^#^	598.68	10	1	2.95	1	142.50	3.81

† Abietanes.

‡ Jatrophanes.

§ Segetanes.

¶ Pepluane.

# Paralianes.

### MDS analysis

**Figure 3.**
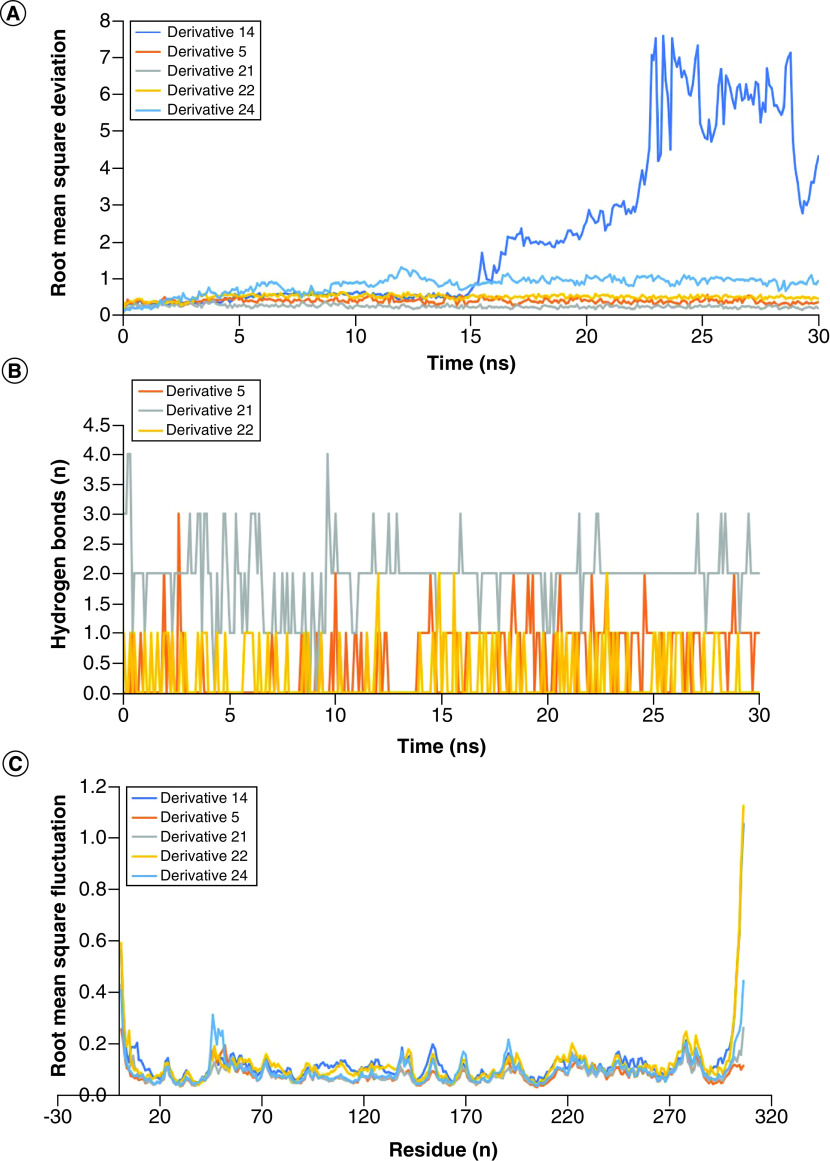
Molecular dynamics simulation data analysis. **(A)** The root mean square deviation values of derivatives bounded with the protein. **(B)** The total number of Hb formed by the derivatives with protein over 30 ns period. **(C)** The root mean square fluctuation values of C-alpha of amino acid residues upon binding of the derivatives. Hb: Hydrogen bond; MDS: Molecular dynamics simulation.

**Figure 4.**
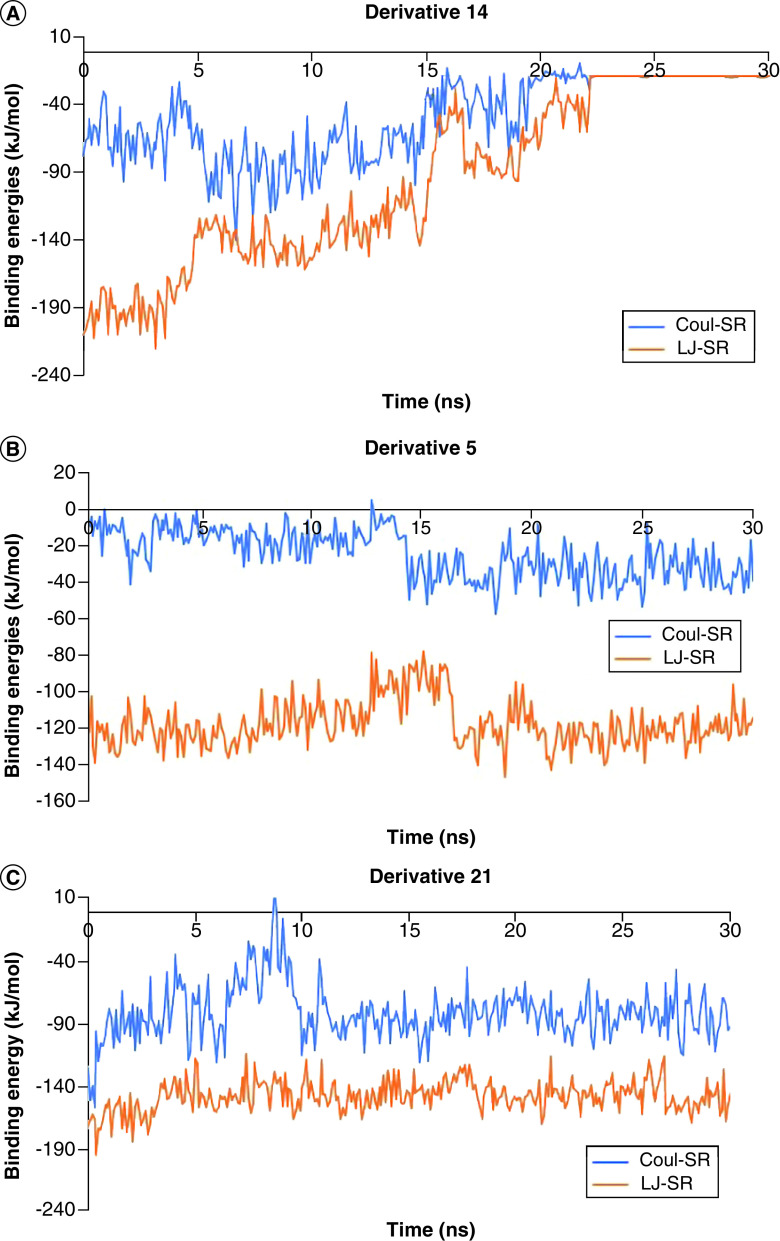

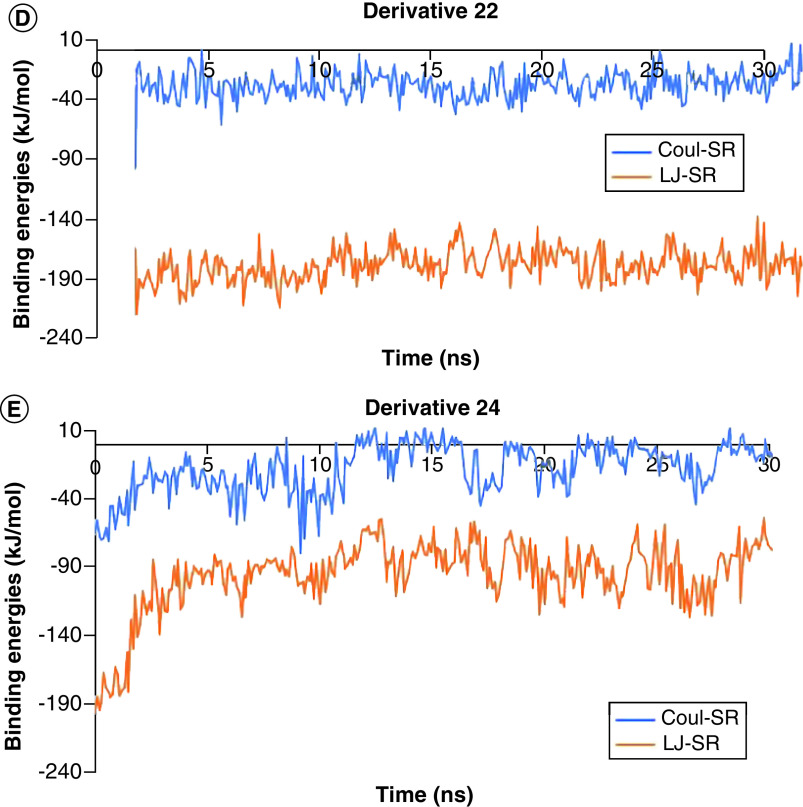
The **Coul-SR and LJ-SR binding energy values of derivatives with the protein.** **(A)** Derivative 14 binding energy values. **(B)** Derivative 5 binding energy values. **(C)** Derivative 21 binding energy values. **(D)** Derivative 22 binding energy values. **(E)** Derivative 24 binding energy values.

**Figure 5.**
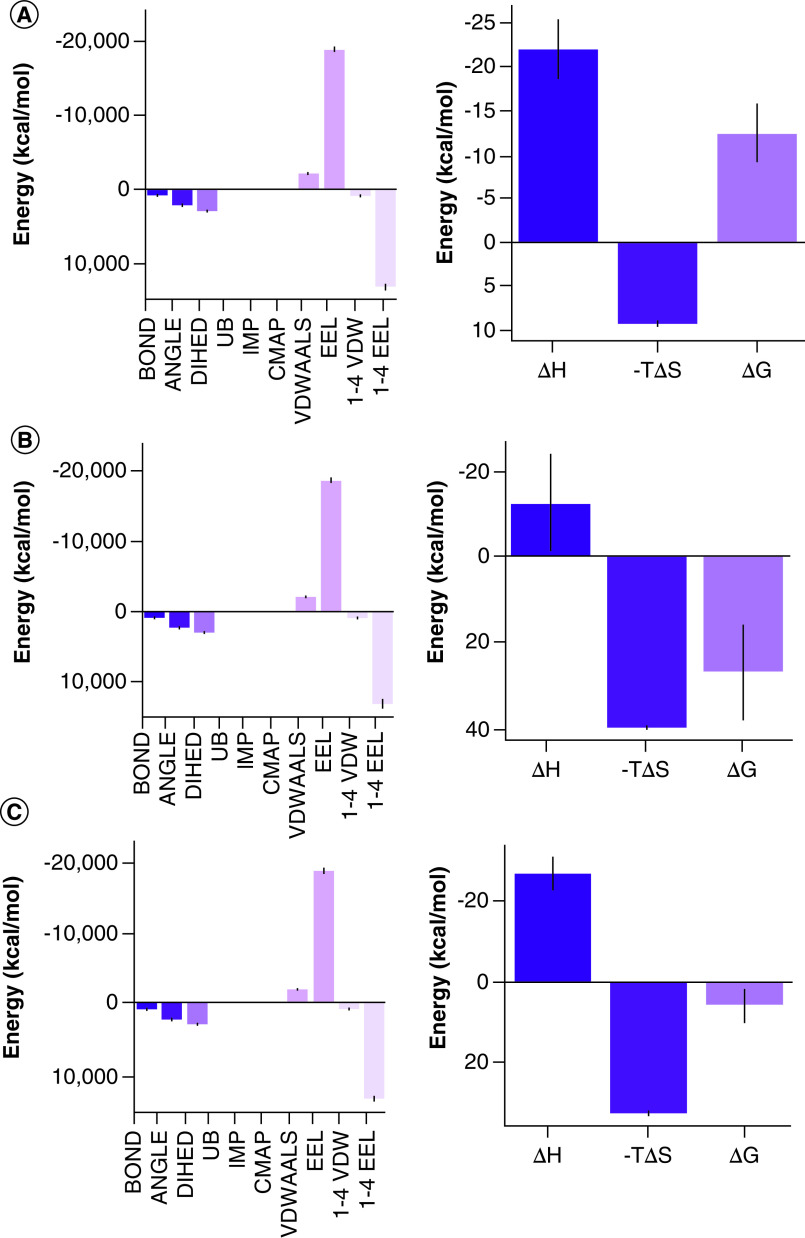

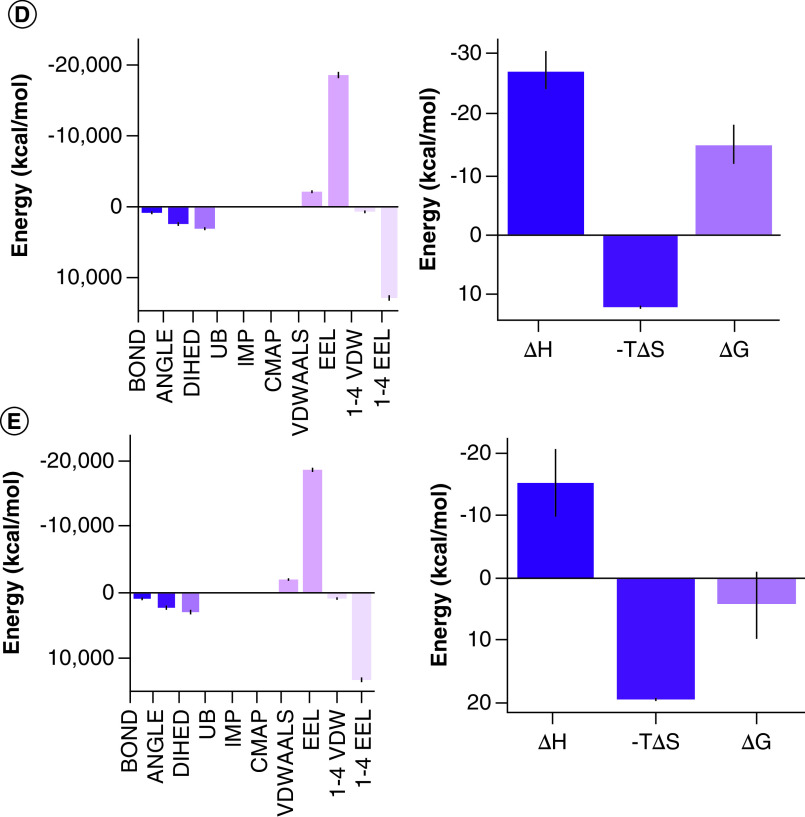
The binding energy values of the five promising derivatives with protein. **(A)** Derivative 5. **(B)** Derivative 14. **(C)** Derivative 21. **(D)** Derivative 22. **(E)** Derivative 24.

The analysis of MD trajectory analysis provided us with detailed insight into the drug-like behavior of the five derivatives. The derivatives 14 and 24 showed higher levels of deviation. This data suggests that these derivatives have changed their position dramatically from their binding site throughout 30 ns as compared with the other three derivatives. On the bases of these observations, we can rule out them as the potential inhibitors of Mpro of SARS-CoV-2. The other three derivatives showed structural deviations lower than 1 Å. This suggests that these are better drug-like candidates [28]. This notion is further strengthened by looking at the total number of hydrogen bonds formed by these derivatives with the protein throughout 30 ns simulations (Figure 3). Furthermore, we also calculated the Coul-SR and LJ-SR interaction energies of the derivatives with the protein. Interestingly, these derivatives showed the lowest interactions energies (Coul-SR = -81.0092 kJ/mol, LJ-SR = -147.51 kJ/mol) (Figure 4). The lowest binding energies also indicate better binding affinities of the derivative with the protein. The binding energies of the derivatives with the Mpro protein were calculated using MMGBSA. Interestingly, derivatives 5 and 22 showed -12.46 kJ/mol and -14.93 kJ/mol, respectively, while the other three derivatives showed positive values (Figure 5). Also, there is a very small confirmation change between the star pose and the last frame pose of MDS for the derivatives 5 and 22 as shown in Supplementary Figures 5 & 6. This data shows that derivatives 5 and 22 can be better drugs to inhibit Mpro protein of COVID-19.

## Discussion

SARS-CoV-2 Mpro consists of three domains. Domain I (residues 8–99) and II (residues 100–183) are six-stranded antiparallel β barrels that resemble the configuration of chymotrypsin and picornavirus 3C proteinases. Domain III (residues 200–300) is a five-helix cluster involved in Mpro proteolytic action. Domain II and domain III are connected by a long loop (residues 184–199). The substrate-binding site of the Mpro, which consists of Cys145 and His41 is located between domains I and II. The oxyanion hole present in the active site consists of the main chain residues Gly143, Ser144 and Cys145. The protein structure and the catalytic dyad are represented in Supplementary Figure 7 [29–31]. Several coronaviral targets, including the Mpro, spike protein, RNA-dependent RNA polymerase (RdRp or nsp12), helicase (nsp13) and PLpro [32,33]. However, the Mpro catalyzes the breakdown of viral polyproteins into almost all replication factors, including nsp12 and nsp13, which are critical for viral replication. Although PLpro participates in processing viral proteins, it contains the ubiquitin recognition motif of deubiquitinases, normally found in the human body. Thus, PLpro inhibitors may also inhibit human deubiquitinases. The polyproteins are cleaved by Mpro at 11 positions, mainly between sequences Leu-Gln and Ser-Ala-Gly; it is reported that no host cell protease cleaves at this specific constriction site [34–36]. In addition, the active site of SARS-CoV-2 Mpro consists of the catalytic dyad Cys144 and His41, while human serine proteinases and other cysteine proteinases contain catalytic triads. Therefore, targeting the Mpro of SARS-CoV-2 is supposed to be selective and cause less toxicity [2,30,35]. Accordingly, SARS-CoV-2 Mpro is a druggable target for potential COVID-19 drugs.

The sequence homology of the Mpro in SARS-CoV-1 and SARS-CoV-2 shared 96–97% similarity [3,37,38], and the active site residues are conserved in both proteins, including the catalytic dyad (His41 and Cys145). Glu166 is the key amino acid that aids in the dimerization of Mpro and formation of the substrate binding pocket; His41 and Cys145, the catalytic dyad, induce changes in the enzyme conformation and chemical features to facilitate hydrolysis of the viral polyproteins. SARS-CoV and SARS-CoV-2 Mpro consist of three domains: two chymotrypsin-like β-domain (I and II) and an α-helical domain [24] enclosing the active site between domains I and II [29,30,38]. As well, the catalytic dyads of the Mpro are superimposed in both coronavirus strains [3]. This high sequence homology and structural similarities could be due to their evolutionary origin and mutual mechanism of pathogenesis.

Our molecular docking findings show that six of the derivatives (5, 13, 14, 21, 22, 24) have higher binding affinities than the standard inhibitor (GC373) for SARS-CoV-2 Mpro. The target protein's active site was identified by studying the crystalized protein-drug complex [39,40]. It was proven that diterpenes have an inhibitory effect on SARS-CoV Mpro, according to the US patent no. CN101418334 (WA, USA) [41]. In addition, abietane-type diterpenes inhibit SARS-CoV 3CLpro [12,13]. Furthermore, several studies confirmed the antiviral activity of diterpenes against SARS-CoV, CHIKV, HIV-1,2, SFV, Sindbis virus and HSV-2 viruses due to the inhibition of essential proteins involved in viral replication [12–14,16]. The Gibbs energy of complex formation was used to calculate the binding affinities of the derivatives with the active site of the protein. Besides, the RMSD value showed a high similarity of the redocked ligand conformation with the experimental ligand conformation in the target protein’s active site.

Lipinski’s rule, one of the most acceptable rules for predicting drug-likeness, was applied to assess the pharmacokinetic properties of the selected derivatives. Lipinski has tested several drugs and identified common physiochemical features among them. Based on Lipinski’s rule, a compound should meet at least three of the following four criteria to have a drug potentiality: no more than 5 hydrogen bond donors (N or O atoms with at least one H atom); no more than 10 hydrogen bond acceptors (N or O atoms); molecular weight of less than 500 Daltons; and Moriguchi octanol–water partition coefficient (Mlog P; lipophilicity) not greater than 4.15 [42]. A drug should also have TPSA (topological surface area; apparent polarity) less than 131.6 Å2 and WLOGP less than 5.88 [43]. Five of the diterpenes derivatives followed Lipinski’s rule and could be a promising potential drug-like candidate.

MDS are frequently used to study dynamic interactions of ligands with proteins [44–46]. Ligand protein docking protocols have several well-documented shortcomings. The binding energy estimations are not realistic in contemporary docking software. Therefore, it is highly recommended to couple the docking studies with MDS. MDS calculate the binding properties and efficacy of ligands using the second law of Newton. This technique provides information about the dynamic behavior of ligand binding with proteins that is not possible in currently available docking algorithms. The MDS data strengthened the docking results and showed that some of the promising compounds have structural deviations lower than 1 Å and their binding with the active site of Mpro was quite stable throughout 30 ns. Also, the small values of binding energies of these derivatives with Mpro suggest strong interaction and potential inhibitory action.

## Conclusion

In this study, we conducted an *in silico* evaluation of promising antiviral diterpenoids to provide molecular insight into the interaction modes with SARS-CoV-2 Mpro. The results indicate that certain diterpenoids of the classes abietane, jatrophane, segetane, pepluane and paraliane have high binding affinities with the protein active site relative to a potent Mpro inhibitor, GC373. In addition, a pharmacokinetic study using SwissADME online tool and MDS analysis showed that some diterpenes have drug-like properties. Our study sheds light on diterpenes as a promising plant-derived compound for future clinical trials for better drug design to combat the COVID-19 pandemic.

**Summary points**

- SARS-CoV-2 main protease (Mpro) showed high sequence homology with SARS-CoV Mpro.
- Some of the diterpenes followed Lipinski’s rule and have the potential to orally active drugs.
- The docking results showed that some of the diterpenes have promising binding affinities with SARS-CoV-2 Mpro due to their unique structural features that enable different electrostatic interaction with the active site of the protein.
- Molecular dynamic simulation data further verified the docking finding and some of the diterpenes derivatives have structural deviations lower than 1 Å, and their binding with the active site of Mpro was quite stable throughout 30 ns.
- This study emphasizes the importance of the plant as a rich source of bioactive components.
