# Supplementary material for: In silico assessment of diterpenes as potential inhibitors of SARS-COV-2 main protease
Source: Future Virol. 2023 May 22;18(5):295–308. doi: 10.2217/fvl-2022-0163 (PMC10207350; doi:10.2217/fvl-2022-0163)
Supplement: Supplementary file 1 [file fvl-18-295-s1.docx]

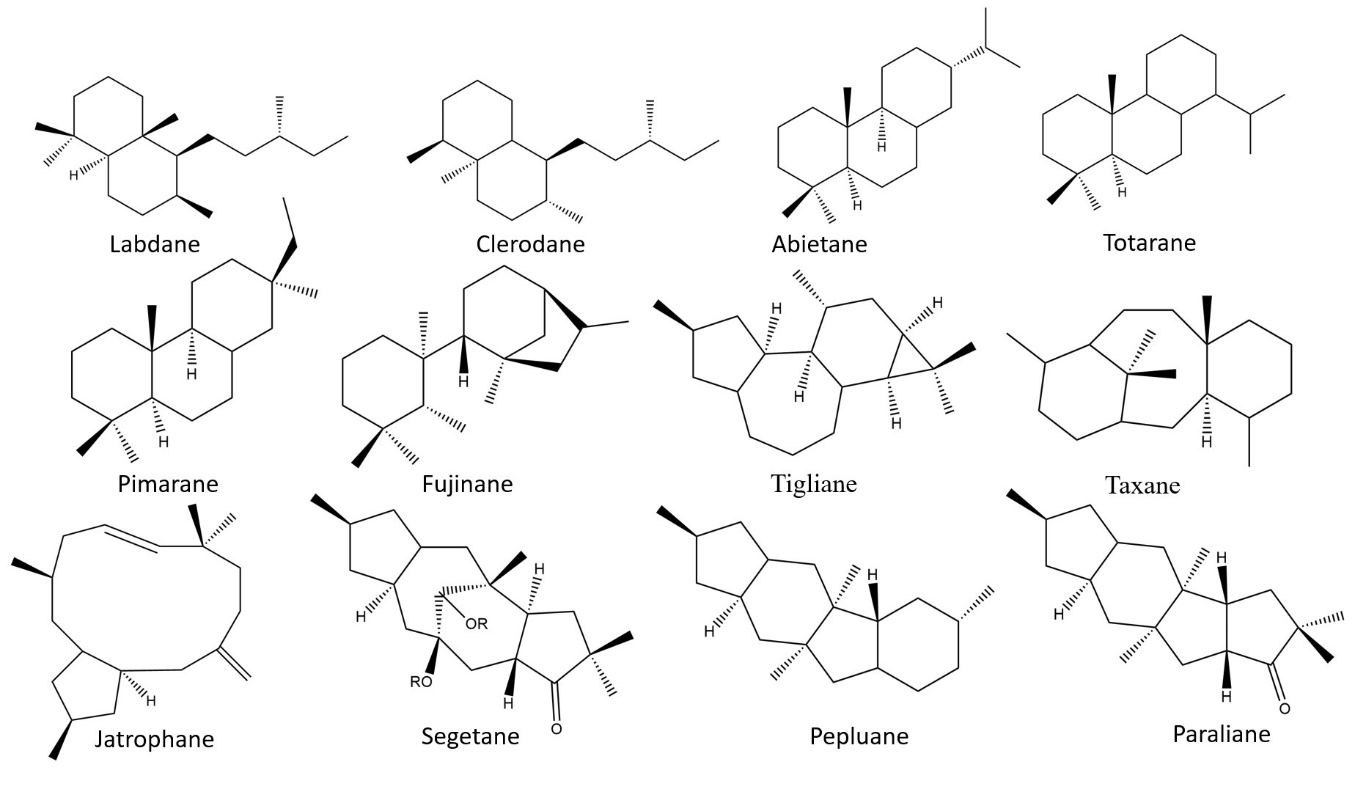


**Figure S1.** The chemical structure of the skeletons of some diterpenes classes.


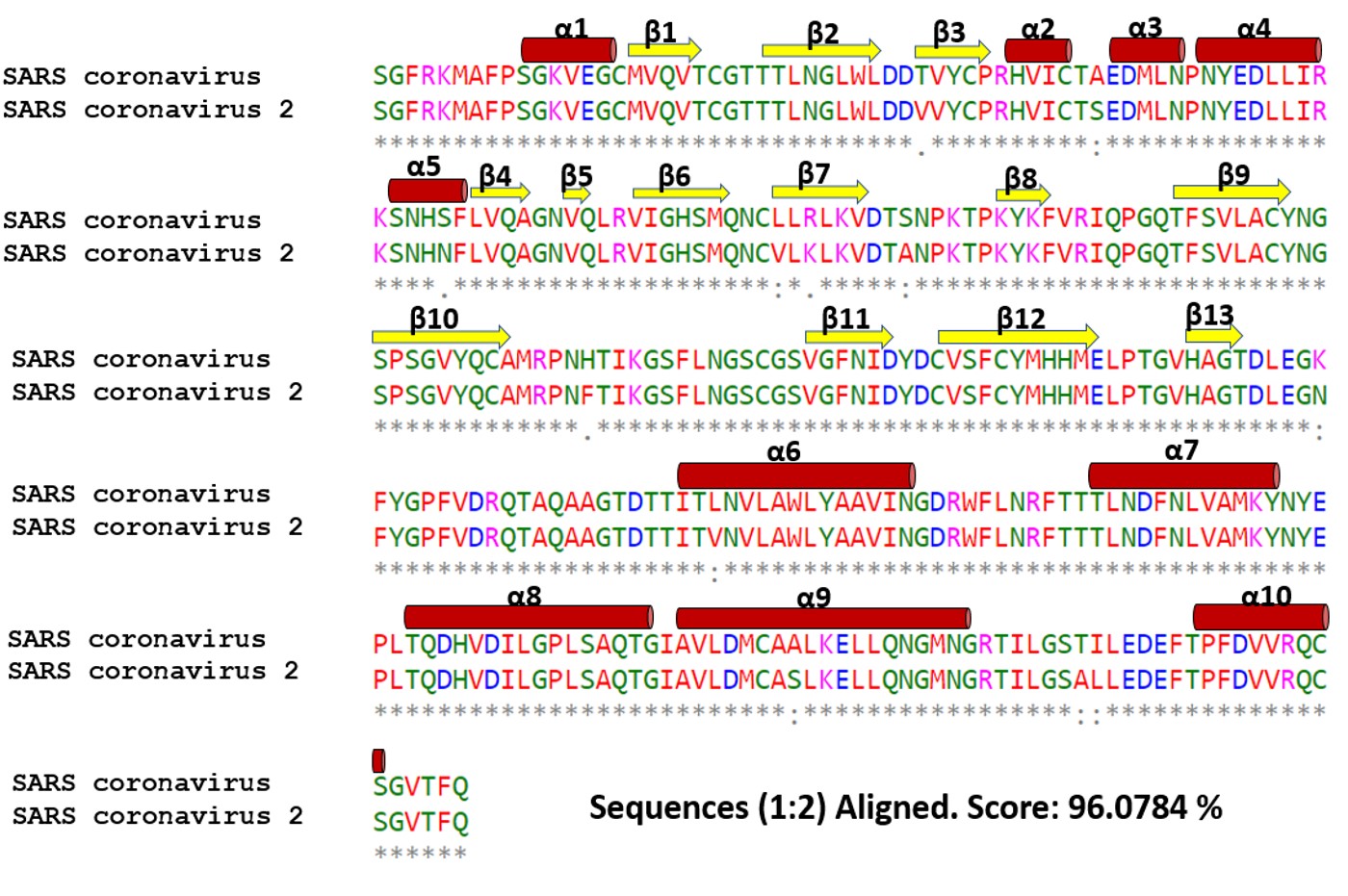


**Figure S2.** The sequence alignment of the SARS-COV Mpro and SARS-COV-2 Mpro and the similarity percentage. The major differences in amino acids are indicated by marks below.


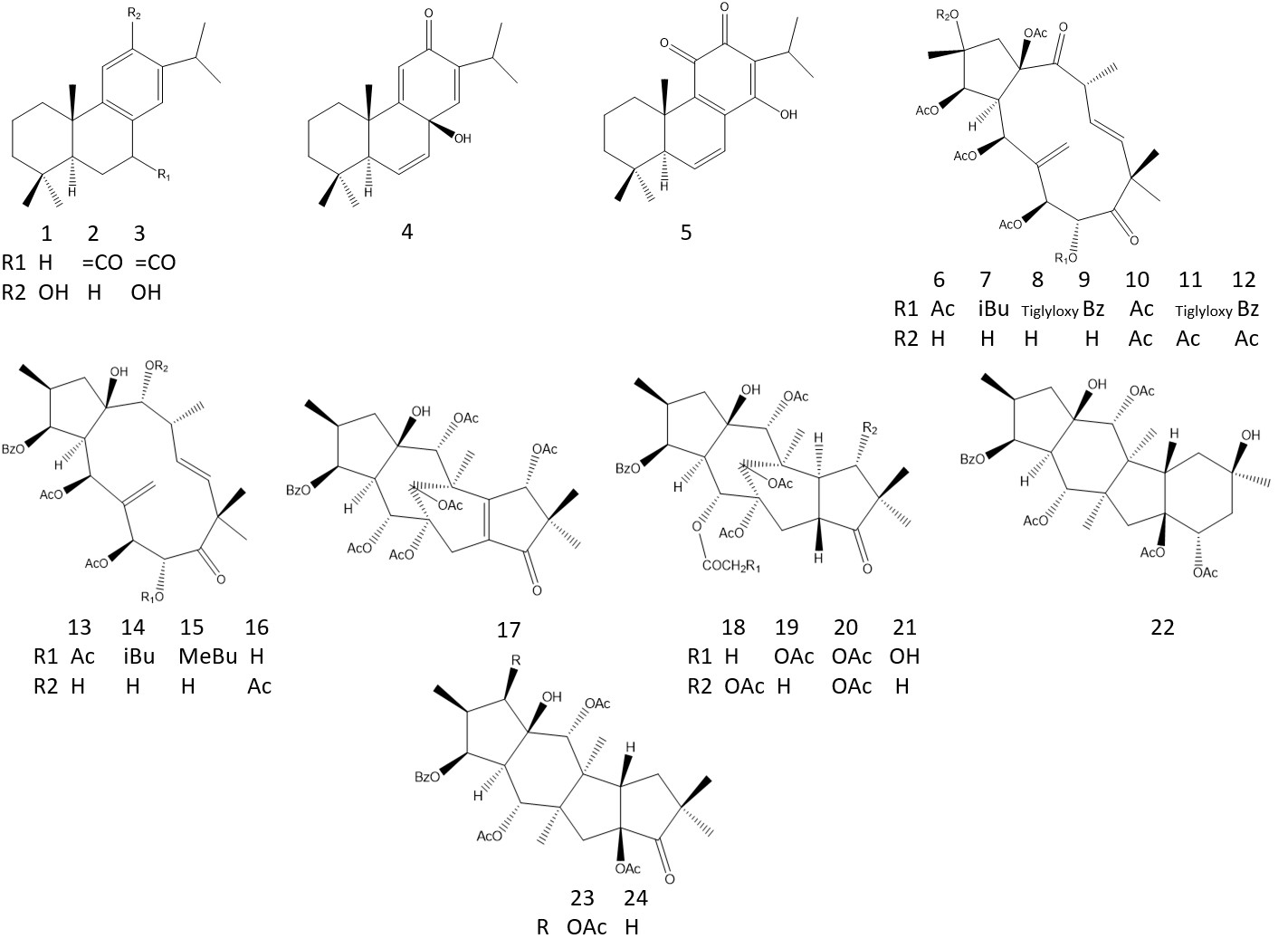


**Figure S3.** The chemical structure of the most important diterpenes derivatives and the standard Mpro inhibitor, GC373. **1-5** abietane derivatives, **6-16** Jatrophane derivatives, **17-21** Segetane derivatives, **22** Pepluane derivative, **23-24** Paraliane derivatives, GC373.


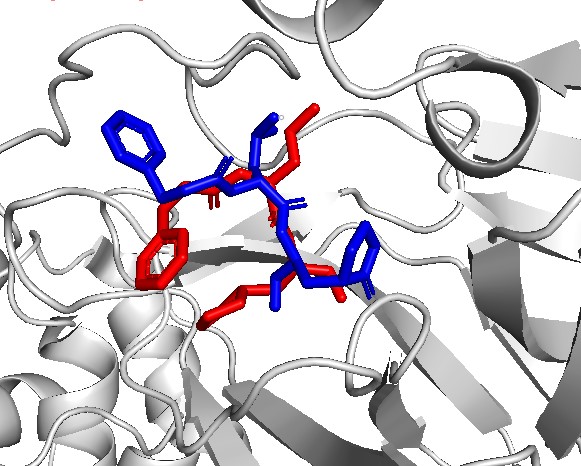


**Figure S4**. 3-D illustration of the superimposed redocked complex GC373-Mpro (blue) on the co-crystallized complex (red).


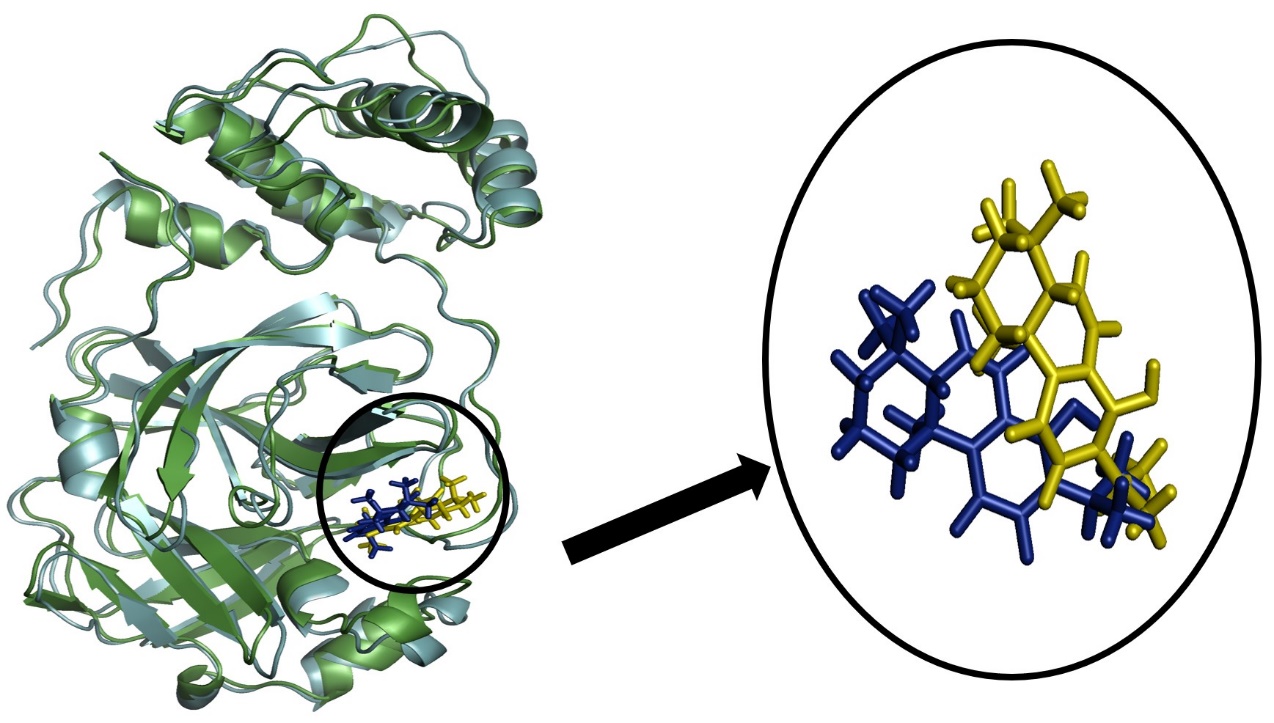


**Figure S5.** Change in confirmation of derivative 5 binding pose with protein. The pose at the start of the simulation is shown in blue, while pose in the last frame of MD simulation is shown in yellow. There is very small conformational change in the derivative pose, suggesting it is a suitable drug to inhibit Mpro of COVID-19.


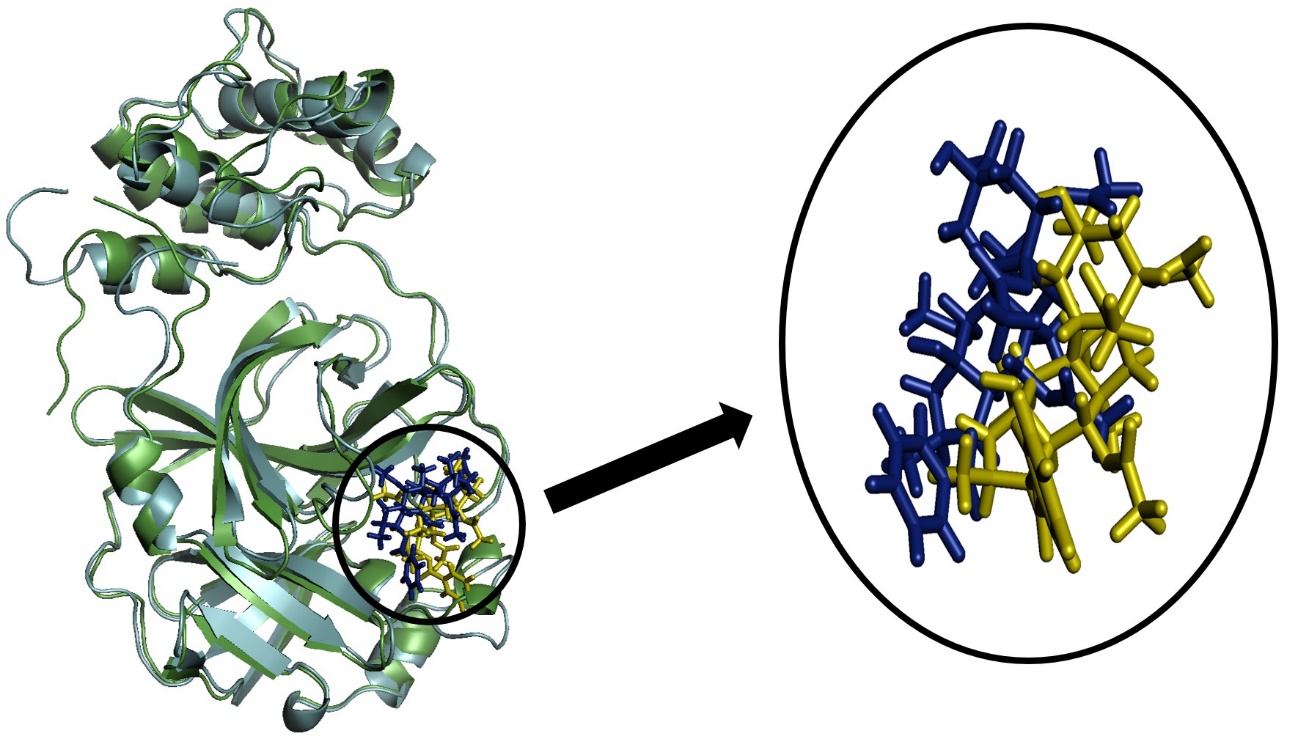


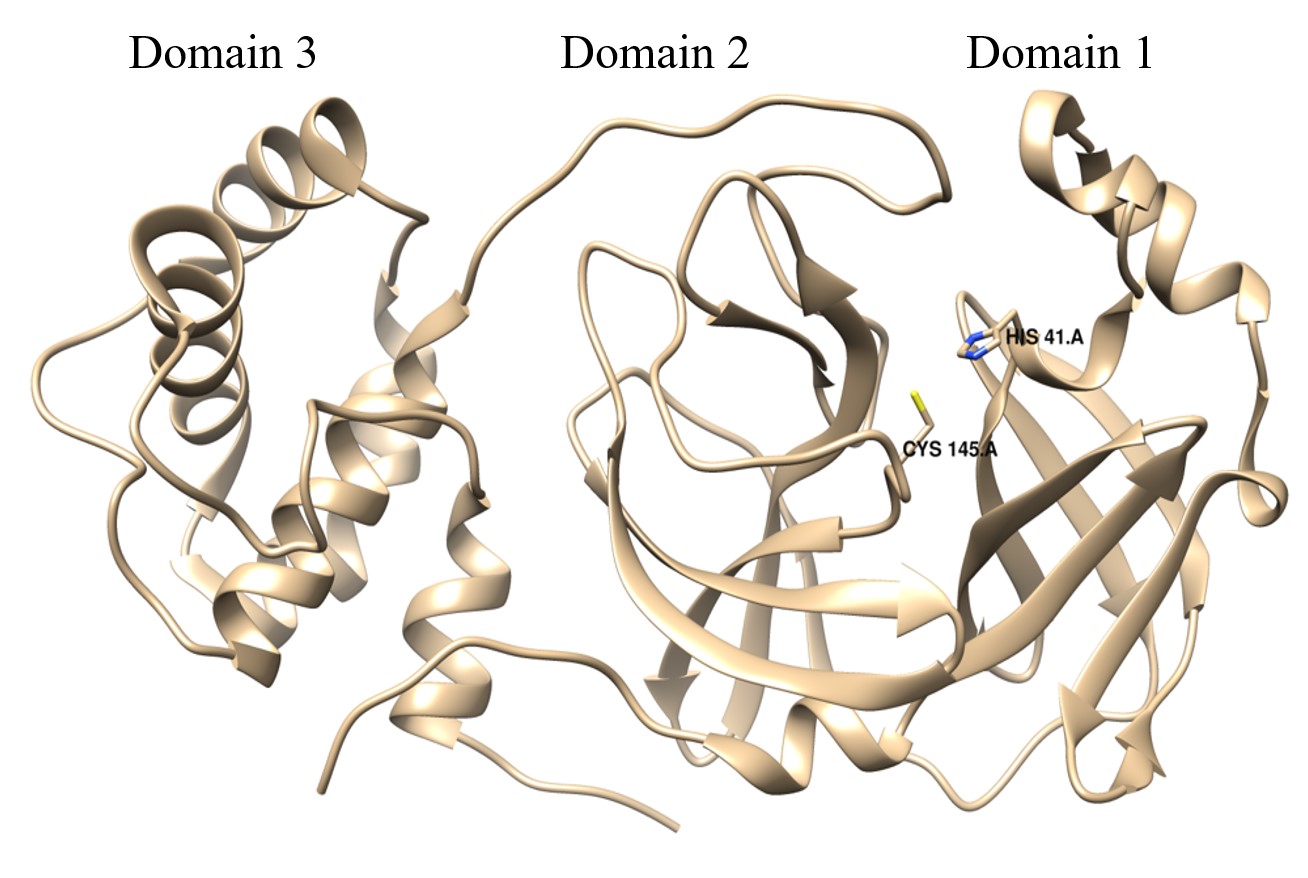
**Figure S6.** Change in confirmation of derivative 22 binding pose with protein. The pose at the start of the simulation is shown in blue, while pose in the last frame of MD simulation is shown in yellow.

**Figure S7.** Apo form of SARS-COV-2 Mpro. The 5 helices on the left represent domain III, the 6 antiparallel β barrels on the right represents domain I and II. Key amino acids, Cys145 and His 41, are found in the substrate binding site between domain I and II.
