# Supplementary material for: In silico assessment of diterpenes as potential inhibitors of SARS-COV-2 main protease
Source: Future Virol. 2023 May 22;18(5):295–308. doi: 10.2217/fvl-2022-0163 (PMC10207350; doi:10.2217/fvl-2022-0163)
Supplement: Supplementary file 2 [file fvl-18-295-s2.docx]

**Table S1.** Antiviral activity of certain derivatives from some classes of diterpenes and their mechanisms of action. ^a^Abietane, ^b^Jatrophane, ^c^Segetane, ^d^Pepluane, ^e^Paraliane derivatives.

| Ref. | Mechanism of action | antiviral activity | Compound | N |
| --- | --- | --- | --- | --- |
| 12,13 | Inhibition of 3CL^PRO^ | SARS coronavirus | Ferruginol | 1^a^ |
| 12,13 | Inhibition of 3CL^PRO^ | SARS coronavirus | dehydroabieta-7-one | 2^a^ |
| 12,13 | Inhibition of 3CL^PRO^ | SARS coronavirus | Sugiol | 3^a^ |
| 12,13 | Inhibition of 3CL^PRO^ | SARS coronavirus | 8β-**hydroxyabieta**-9(11),13-dien-12-one | 4^a^ |
| 12,13 | Inhibition of 3CL^PRO^ | SARS coronavirus | 6,7-dehydroroyleanone | 5^a^ |
| 14 | Inhibition of replication via protein kinase C isozymes modulation | CHIKV | (2R,3R,4S,5R,7S,8R,13R,15R)-3,5,7,8,15-pentaacetoxy-2-hydroxy-9,14-**dioxojatropha**-6(17)-11E-diene | 6^b^ |
| 14 | Inhibition of replication via protein kinase C isozymes modulation | CHIKV, HIV-1,2, SFV, Sindbis virus | (2R,3R,4S,5R,7S,8R,13R,15R)-3,5,7,15-tetraacetoxy-2-hydroxy-8-isobutoxy-9,14-**dioxojatropha**-6(17)-11E-diene | 7^b^ |
| 14 | Inhibition of replication via protein kinase C isozymes modulation | CHIKV, HIV-1,2, SFV, Sindbis virus | (2R,3R,4S,5R,7S,8R,13R,15R)-3,5,7,15-tetraacetoxy-2-hydroxy-8-tiglyloxy-9,14-**dioxojatropha**-6(17)-11E-diene | 8^b^ |
| 14 | Inhibition of replication via protein kinase C isozymes modulation | CHIKV, HIV-1,2, SFV, Sindbis virus | (2R,3R,4S,5R,7S,8R,13R,15R)-3,5,7,15-tetraacetoxy-2-hydroxy-8-benzoyloxy-9,14-**dioxojatropha**-6(17)-11E-diene | 9^b^ |
| 14 | Inhibition of replication via protein kinase C isozymes modulation | CHIKV, HIV-1,2, SFV, Sindbis virus | (2R,3R,4S,5R,7S,8R,13R,15R)-2,3,5,7,8,15-hexaacetoxy-9,14-**dioxojatropha**-6(17)-11E-diene | 10^b^ |
| 14 | Inhibition of replication via protein kinase C isozymes modulation | CHIKV, HIV-1,2, SFV, Sindbis virus | (2R,3R,4S,5R,7S,8R,13R,15R)-2,3,5,7,15-pentaaacetoxy-8-tiglyloxy-9,14-**dioxojatropha**-6(17)-11E-diene | 11^b^ |
| 14 | Inhibition of replication via protein kinase C isozymes modulation | CHIKV HIV-1,2, SFV, Sindbis virus | (2R,3R,4S,5R,7S,8R,13R,15R)-2,3,5,7,15-pentaacetoxy-8-benzoyloxy-9,14-**dioxojatropha**-6(17)-11E-diene | 12^b^ |
| 14 | Inhibition of replication via protein kinase C isozymes modulation | CHIKV, HIV-1,2, SFV, Sindbis virus | (2R,3R,4S,5R,7S,8R,13R,14S,15R)-5,7,8-triacetoxy-3-benzoyloxy-14,15-dihydroxy-9-**oxojatropha**-6(17)-11E-diene | 13^b^ |
| 14 | Inhibition of replication via protein kinase C isozymes modulation | CHIKV, HIV-1,2, SFV, Sindbis virus | (2R,3R,4S,5R,7S,8R,13R,14S,15R)-5,7-diacetoxy-3-benzoyloxy-8-isobutoxy-14,15-dihydroxy-9-**oxojatropha**-6(17)-11E-diene | 14^b^ |
| 14 | Inhibition of replication via protein kinase C isozymes modulation | CHIKV, HIV-1,2, SFV, Sindbis virus | (2R,3R,4S,5R,7S,8R,13R,14S,15R)-5,7-diacetoxy-3-benzoyloxy-8-(pentan-2-yloxy)-14,15-dihydroxy-9-**oxojatropha**-6(17)-11E-diene | 15^b^ |
| 14 | Inhibition of replication via protein kinase C isozymes modulation | CHIKV, HIV-1,2, SFV, Sindbis virus | (2R,3R,4S,5R,7S,8R,13R,14S,15R)-5,7,14-triacetoxy-3-benzoyloxy-8,15-dihydroxy-9-**oxojatropha**-6(17)-11E-diene | 16^b^ |
| 15 | Inhibition of replication | HIV-1 | (2S,3S,4R,5R,6R,11R,13S,14R,15R,16R)-5,11,14-triacetoxy-3-benzoyloxy-15-hydoxy-**segetan**-8-ene | 17^c^ |
| 15 | Inhibition of replication | HIV-1 | (2S,3S,4R,5R,6R,8R,11R,12S,13S,14R,15R,16R)-5,11,14-triacetoxy-3-benzoyloxy-15-hydoxy-**segetane** | 18^c^ |
| 15 | Inhibition of replication | HIV-1 | (3S,4R,5R,6R,8R,11R,12S,13R,14R,15R,16R)-5-(2-acetoxyacetoxy)-14-acetoxy-3-benzoyloxy-15-hydoxy-**segetane** | 19^c^ |
| 15 | Inhibition of replication | HIV-1 | (2S,3S,4R,5R,6R,8R,11S,12S,13R,14R,15R,16R)-5-(2-acetoxyacetoxy)-11,14-diacetoxy-3-benzoyloxy-15-hydoxy-**segetane** | 20^c^ |
| 15 | Inhibition of replication | HIV-1 | (2S,3S,4R,5R,6R,8R,12S,13R,14R,15R,16R)-14-acetoxy-3-benzoyloxy-5-(2-hydroxyacetoxy)-15-hydoxy-**segetane** | 21^c^ |
| 16 | n.d. | HSV-2 | (2S,3S,4R,5R,6R,8S,9S,11R,13S,14S,15R,16R)-5,8,9,15-tetraacetoxy-3-benzoyloxy-11,16-dihydroxy-**pepluane** | 22^d^ |
| 15 | Inhibition of replication | HIV-1 | (1R,2S,3R,4R,5R,6R,8S,12S,13S,14R,15R)-1,5,8,14-tetraacetoxy-3-benzoyloxy-15-hydroxy-**paraliane** | 23^e^ |
| 15 | Inhibition of replication | HIV-1 | (2S,3R,4R,5R,6R,8S,12S,13S,14R,15R)-5,8,14-triacetoxy-3-benzoyloxy-1,15-dihydroxy-**paraliane** | 24^e^ |
